# Motor Skills: Recruitment of Kinesins, Myosins and Dynein during Assembly and Egress of Alphaherpesviruses

**DOI:** 10.3390/v13081622

**Published:** 2021-08-17

**Authors:** Duncan W. Wilson

**Affiliations:** 1 Department of Developmental and Molecular Biology, Albert Einstein College of Medicine, 1300 Morris Park Avenue, Bronx, NY 10461, USA; duncan.wilson@einsteinmed.org; Tel.: +1-718-430-2305; 2 Dominick P. Purpura Department of Neuroscience, Albert Einstein College of Medicine, 1300 Morris Park Avenue, Bronx, NY 10461, USA

**Keywords:** herpes simplex virus, pseudorabies virus, kinesin, dynein, myosin, microtubules, actin

## Abstract

The alphaherpesviruses are pathogens of the mammalian nervous system. Initial infection is commonly at mucosal epithelia, followed by spread to, and establishment of latency in, the peripheral nervous system. During productive infection, viral gene expression, replication of the dsDNA genome, capsid assembly and genome packaging take place in the infected cell nucleus, after which mature nucleocapsids emerge into the cytoplasm. Capsids must then travel to their site of envelopment at cytoplasmic organelles, and enveloped virions need to reach the cell surface for release and spread. Transport at each of these steps requires movement of alphaherpesvirus particles through a crowded and viscous cytoplasm, and for distances ranging from several microns in epithelial cells, to millimeters or even meters during egress from neurons. To solve this challenging problem alphaherpesviruses, and their assembly intermediates, exploit microtubule- and actin-dependent cellular motors. This review focuses upon the mechanisms used by alphaherpesviruses to recruit kinesin, myosin and dynein motors during assembly and egress.

## 1. Introduction 

### 1.1. The Alphaherpesvirinae

**Figure 1:**
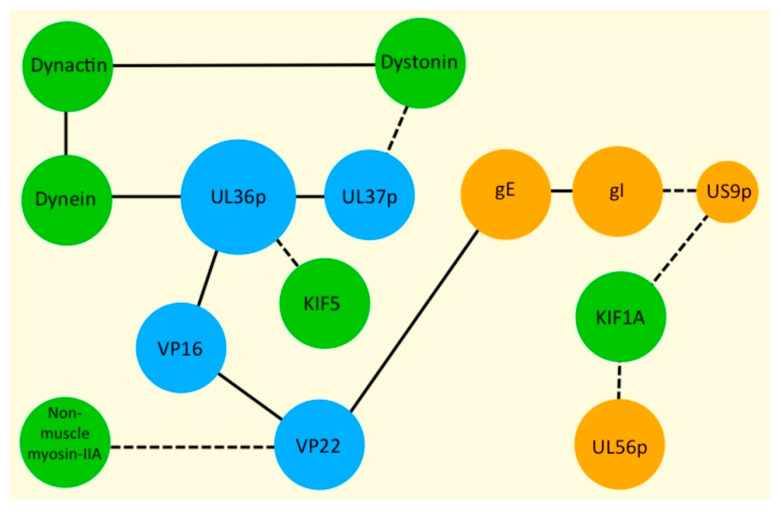
Interactions between alphaherpesvirus and host-cell proteins. Virally encoded tegument and membrane-imbedded proteins are shown in blue and orange, respectively. Host-cell proteins, or protein complexes, are in green. Solid lines indicate direct protein-protein interactions known to occur in alphaherpesvirus-infected cells. Broken lines represent interactions that may be indirect, or where a proposed protein–protein interaction has yet to be demonstrated in the context of a virally infected cell. See text for details.

The *Alphaherpesvirinae* include pathogens of the nervous system such as herpes simplex virus types 1 and 2 (HSV-1 and HSV-2), varicella zoster virus (VZV) and the swine virus pseudorabies virus (PRV) [1,2,3,4]. Many alphaherpesvirus infections originate at peripheral locations, such as epithelial tissues, then spread to adjacent neurons of the peripheral nervous system to establish lifelong latency [5,6]. In addition to causing oral and genital lesions, HSV infections can result in keratoconjunctivitis (the leading cause of non-traumatic corneal blindness in the United States) or fatal encephalitis [2], and the virus can be transmitted in utero and during or after birth, resulting in neonatal infections of the skin, eyes, mouth, internal organs and central nervous system [7]. HSV-1 infection of the brain may also contribute to the progression of Alzheimer’s disease, though this issue remains controversial [8,9,10,11]. The alphaherpesviruses are complex particles composed of about 40 structural proteins distributed between three distinct layers: (i) An icosahedral capsid packaged with the dsDNA genome, which is ~150 kb in size in the case of HSV-1, HSV-2 and PRV, (ii) A complex multi-subunit protein layer termed tegument, that is anchored to the capsid surface, (iii) An envelope, derived from the lipid bilayer of a host cell cytoplasmic organelle, which contains numerous virally encoded membrane proteins. The structure and composition of HSV-1 particles have recently been reviewed in detail [12,13,14,15,16,17]. Table 1 lists alphaherpesvirus proteins discussed in this review, and Figure 1 summarizes relevant interactions between viral and host-cell proteins.

Assembly and egress of alphaherpesviruses requires a complex interplay between viral structural components and the host cell biosynthetic machinery, organelles and cytoskeleton [6,12,15,17]. This review focuses upon the ways in which alphaherpesvirus particles recruit the cellular motors kinesin, dynein and myosin during viral maturation and exit from the infected cell. 

### 1.2. Overview of the Cellular Motors Kinesin, Dynein and Myosin

**Figure 2:**
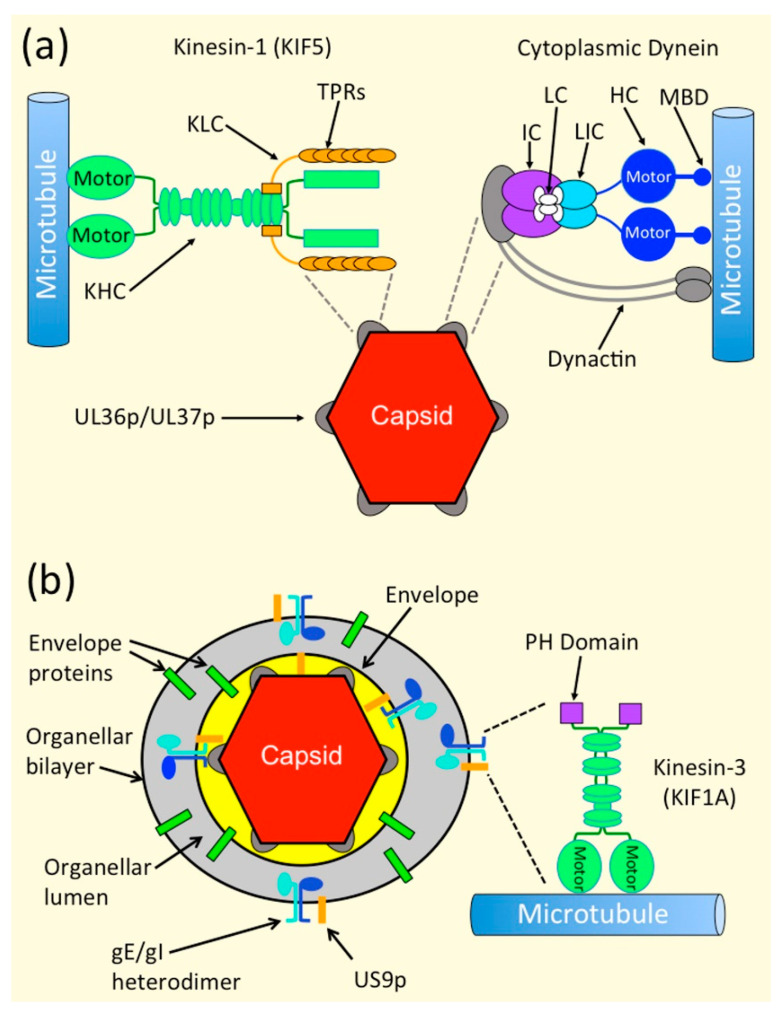
(**a**) During egress, but prior to cytoplasmic envelopment, the alphaherpesvirus capsid (red hexagon) recruits the motors kinesin-1 (left) and dynein/dynactin (right) for association with MTs (blue cylinders). The motors may bind via the inner tegument proteins UL36p/UL37p (grey ovals) at the capsid vertices. Left of panel: Kinesin-1 KHCs are shown in green, and the motor domain is indicated. Ovals represent coiled-coil domains, circles represent hinge regions. The KLCs, with their TPRs, are shown in orange (adapted from [53]). Right of panel: Dynein HC is shown in dark blue, and motor region and MT-binding domain (MBD) are indicated. Additional subunits are the intermediate chains (IC), light intermediate chains (LIC) and light chains (LC). The dynactin complex is shown in grey. Although dynein commonly binds cargo via dynactin, this may not necessarily be the case for HSV-1 capsids. Broken grey lines indicate that, while UL36p/UL37p may recruit kinesin-1 and dynein during egress, the molecular details are unknown; (**b**) The organelle-associated enveloped virion (OEV), following capsid cytoplasmic envelopment. Capsid, with UL36p/UL37p inner tegument is shown as in (**a**). Outer tegument is shown in yellow. The mature, enveloped alphaherpesvirus particle resides within the lumen (grey) of its envelopment organelle. Multiple virally encoded membrane proteins (green bars) are imbedded in the viral envelope and bounding organellar membrane. The gE/gI heterodimer is shown as light and dark blue lollipops, and US9p as an orange bar. Broken grey lines indicate that gE/gI-US9p in the organellar bilayer recruits kinesin KIF1A, shown at right. The activated KIF1A motor is a homodimer [green chains, with coiled-coils and motor domains, as shown for the KHC in (**a**)]. Pleckstrin homology (PH) domains are indicated by purple squares. See text for more details.

Alphaherpesvirus assembly and egress requires transport of viral particles from the vicinity of the infected cell nucleus to the cell periphery. It is therefore inevitable that kinesin motors play a major part in these events (Section 1.2.1). Nevertheless, as detailed in this review, dynein and myosin motors also play important roles in egress and transmission of these viruses, in less well understood ways (Figure 2). 

#### 1.2.1. The Kinesin Superfamily 

There are 45 known kinesins in the mammalian genome [53,54,55,56], most of which transport cargo to the plus-ends of microtubules (MTs), and thus from central regions of the cell to the periphery, and along neuronal axons to the nerve terminal [53,54,55,56]. Kinesins exhibit overlapping cargo specificity, and a variety of mechanisms for cargo-binding [54]. A given cargo can also simultaneously bind more than one type of kinesin motor, with complex outcomes for its trafficking properties [53,57,58]. Very little is known of how and why cargo proteins, organelles and viruses select particular kinesins, or combinations of kinesins, for their transport [53,55,56,59,60]. As discussed in this review (Section 3 and Section 4), evidence suggests that alphaherpesviruses favor kinesin-1 family members, and the kinesin-3 KIF1A, for MT-directed transport during egress [6,61,62,63] (Figure 2). 

Kinesin-1 is a heterotetramer consisting of two kinesin heavy-chains (KHC) and two kinesin light-chains (KLC) [53,54,55,56] (Figure 2). The KHC contains an N-terminal motor domain, followed by a neck and coiled-coil stalk that mediates KHC dimerization, and a globular C-terminal tail. Three KHC isoforms exist, encoded by the KIF5A, KIF5B and KIF5C genes. KIF5B is ubiquitously expressed while KIF5A and KIF5C are largely neuron-specific [53,54,55,56], and each KHC isoform only assembles into homodimers [53]. A pair of KLCs bind to the KHC dimer, close to the end of the KHC coiled-coil stalk. Each KLC contains six tetratricopeptide repeat (TPR) motifs, that can mediate attachment of the tetrameric kinesin-1 to cargo [64,65,66]. In neurons, kinesin-1 localizes to, and preferentially traffics along, axonal microtubules that are non-dynamic, stabilized and bundled as a result of α-tubulin acetylation at residue K_40_ [67,68,69]. Indeed, during development, kinesin-1 accumulates only within neurites destined to become axons, not dendrites [70,71], a phenomenon that may actually drive axon specification [72]. Kinesin-1 is therefore well suited for MT-directed anterograde traffic along axons, and transports a variety of cargo including mitochondria, lysosomes, synaptic vesicle precursors containing synaptotagmin and synaptobrevin, and vesicles carrying presynaptic membrane proteins such as syntaxin 1 and SNAP25 [54,55]. 

Kinesin-3 motors [53,54,55,56], including KIF1A, exist as inactive soluble monomers when not bound to cargo [53,73]. Binding to the lipid bilayer of a cargo vesicle increases the local concentration of the kinesin-3 chains, favoring intermolecular association and driving dimerization [73]. Association of KIF1A with bilayers is facilitated by a C-terminal pleckstrin homology (PH) domain [74], which binds phosphatidylinositol 4,5-bisphosphate (PI [4,5] P_2_) [53,54,55,56] (Figure 2). Cargo-loaded, dimeric kinesin-3 motors are fast (~1–2.5 µm s^−1^) [73], and exhibit “superprocessive” motion with average run lengths of ~10 μm, nearly 10 times that of kinesin-1 [73]. Kinesin-3 motors therefore appear to have evolved to drive long-distance intracellular and axonal transport, and have been termed the “marathon runners” of the cellular world [73]. KIF1A motors traffic synaptic vesicle precursors containing synaptophysin, synaptotagmin, and Rab3A from neuronal cell bodies, along axons, to the nerve terminus [54,55].

#### 1.2.2. Cytoplasmic Dynein 

Cytoplasmic dynein is a MT minus end-directed motor of approximately 1.5 mDa, containing multiple subunits [55]. Two heavy chains (HC) of ~520 kDa contain the MT-binding domains (MBD), connected by stalks to the AAA-ATPase motor domains. The HCs form a complex with two intermediate chains (IC), four light intermediate chains (LIC) and several light chains (LC) (Figure 2). These chains play roles in dynein assembly, stability and cargo binding, and the N-terminal regions of the IC subunits provide a scaffold for interaction between dynein and its partner dynactin [55,75]. Dynactin is a multisubunit complex that is required for most types of dynein-based motility in vivo. It binds to dynein and MTs, can serve as an adaptor linking dynein to cargo, and enhances dynein processivity [55,75] (Figure 2). Like kinesins, dynein recognizes a broad spectrum of cargo, but does so by recruiting alternative dynein subunits and dynactin complexes [55,75]. As might be expected, dynein plays important roles in retrograde transport during alphaherpesvirus entry and infection in neuronal and non-neuronal cells [6,76,77,78,79,80]. However, as detailed in this review, dynein is also present on alphaherpesvirus particles at various stages during their egress (Section 3.5 and Section 4.5). 

#### 1.2.3. The Myosin Superfamily

The human genome contains 40 different myosin genes, encoding 20 distinct classes of myosin [81,82]. Myosins are typically identified by roman numerals, with muscle-associated myosin-II the prototypical family member. Muscle myosin-II (M-II), and a non-muscle form of myosin-II (NM-II) are termed “conventional myosins” and all other family members “unconventional myosins” [81,82,83]. All myosins travel along actin filaments to carry out their functions, using their N-terminal actin-binding motor domain. The tails are quite variable, allowing for diverse functions including cargo binding and anchoring of the myosin chain to specific sites for movement relative to actin [82]. Alpha-helical regions in the tail can also drive coiled-coil formation and dimerization of some myosin family members. As we describe in this review, isoforms of NM-II and myosin-V have been implicated in traffic of alphaherpesvirus viral particles within and between infected cells (Section 2 and Section 5). 

NM-II, like M-II, comprises a myosin chain dimer in complex with additional polypeptides involved in regulation and stability [81]. Dozens of these complexes then bundle together to form thick bipolar filaments. These thick myosin fibers interdigitate with filamentous actin (F-actin), then slide past each other as a result of repetitive interactions between the myosin heads and the actin. This generates the force needed for muscular contraction (M-II) or cytokinesis (NM-II) [81,82,84]. NM-II also plays fundamental roles in cell adhesion and cell migration [84]. Vertebrates express two NM-II isoforms, NM-IIA and -IIB, at similar levels in most cell types [85].

Myosin-V exists as three isoforms in mice and humans: Va, Vb and Vc. The former two are widely expressed in the CNS, whereas Vc appears to be epithelial cell-specific [86]. The myosin-V chain forms a dimer, each bound to six regulatory calmodulin light chains [86]. The myosin-Va and Vb motors are highly processive, and transport a wide variety of protein and organellar cargo long distances in neurons [86]. Myosin Va has also been observed in Tunneling nanotubes (TNTs) formed by astrocytes [87], and may play a role in transport of endocytic organelles within these structures [88,89] (Section 5). 

## 2. Transport of Newly Assembled, Packaged Capsids through the Nucleoplasm to the Perinuclear Space

Following viral gene expression and DNA replication in the infected cell nucleus, the alphaherpesvirus genome is packaged into preassembled procapsids [2,12,16]. Mature, packaged capsids then traverse the nucleoplasm to reach the inner nuclear membrane, where the UL31p/UL34p nuclear export complex [90,91] drives budding into the perinuclear space to generate primary enveloped virions [92,93,94]. Several lines of evidence raised the possibility that capsid transport within the nucleoplasm might be mediated by an actin–myosin-dependent mechanism [95]. In infected human epithelial type 2 (HEp-2) cells, intranuclear HSV-1 capsids exhibited ~0.2–1 µm s^−1^ radial motion [95] around structures interpreted to be assemblons (aggregates of immature intranuclear capsids [96]) and this motion was sensitive to ATP depletion and to the myosin inhibitor 2,3-butanedione monoxime (BDM) [95]. However, these data were interpreted with caution since BDM is known to have broad effects, including upon non-myosin proteins [95,97], and because although capsid motion was sensitive to the actin depolymerizing drug latrunculin A, it was insensitive to actin depolymerization by cytochalasin D. Intranuclear actin filaments, located at the periphery of assemblons and that were also immunostained for myosin-V, were also reported in PRV-infected rat superior cervical ganglia (SCG) [98]. However, subsequent studies in infected mouse embryonic fibroblasts (MEFs) detected no F-actin in the nucleus, even though intranuclear transport of capsids occurred during infection by HSV-1, PRV, Mouse Cytomegalovirus (a Betaherpesvirus) and Murine Gammaherpesvirus 68 [99]. Moreover, as had been previously observed for the actin depolymerizing drug cytochalasin D [95], jasplakinolide (which destroys physiological F-actin structures) and mycalolide B (which severs F-actin) did not inhibit intranuclear capsid transport [99]. Further investigation revealed that latrunculin A, the one actin depolymerizing drug that inhibited intranuclear HSV-1 capsid transport [95], induced the formation of thick actin rods within the interior of the nucleus [99]. Immobile capsids accumulated around these actin rods, and immunoprecipitation experiments suggested the capsids had become bound and trapped by them. Consistent with this explanation, intranuclear capsid motility was unaffected by latrunculin-A in porcine kidney epithelial (PK15) cells, which did not accumulate intranuclear actin rods in the presence of the drug [99]. Most recently, studies in male rat kangaroo epithelial (PtK2) cells suggest that nuclear HSV-1 and PRV capsids do not use a motor-directed transport mechanism. Instead, it has been proposed that the increased volume of interchromatin domains during infection is sufficient to ensure delivery of capsids to the nuclear envelope by diffusion alone [100]. 

## 3. Transport of Cytoplasmic Capsids to Their Site of Envelopment

### 3.1. Emergence of Capsids into the Cytoplasm and Recruitment of Tegument 

Primary enveloped virions in the perinuclear space undergo fusion of their envelopes with the outer nuclear membrane to deliver capsids into the cytoplasm. Capsids then utilize kinesin motors for transport to cytoplasmic organelles for secondary envelopment [12,14,15,17,21]. It is thought that attachment of kinesins to cytoplasmic capsids is mediated by components of the tegument [12,13,17,101,102], specifically the inner tegument proteins UL36p and its binding partner UL37p (Table 1, Figure 1 and Figure 2). 

UL36p is a ~330 kDa polypeptide anchored to the vertices of the icosahedral capsid [12,13,17,103], while UL37p is an ~120 kDa protein with an amino terminal portion resembling cellular multisubunit tethering complexes (MTCs) that help dock transport vesicles to their target membranes [27,28,104,105,106,107,108] (Figure 2). UL36p and UL37p perform multiple functions including capsid envelopment in the cytoplasm [12,17,20,30,109], recruitment of outer tegument polypeptides [12,13,110,111] and, as detailed in Section 3.2, Section 3.3, Section 3.4 and Section 3.5, motor recruitment. UL36p and UL37p become attached to alphaherpesvirus capsids in the nucleoplasm or soon after their entry into the cytoplasm ([112,113,114,115,116,117,118], recently reviewed in [17]). This ensures that, immediately upon their export from the nucleus, capsids are poised to recruit kinesin motors and begin their journey through the cytoplasm [12,21]. 

### 3.2. Loss of the UL36 and UL37 Genes Disrupt Capsid Trafficking in the Cytoplasm

Live-cell imaging of Vero cells infected by HSV-1 expressing a fluorescently tagged capsid, revealed that 10–30% of cytoplasmic capsids moved dynamically over long distances, with a mean velocity of ~2 µm s^−1^ [21]. In contrast, HSV-1 mutants deleted for either UL36 or UL37 moved at a significantly lower velocity than the parental control strain, and exhibited only undirected oscillatory movements with frequent changes in direction, resembling random diffusion [21]. Similar results were obtained for the transport of UL36- and UL37-null HSV-1 capsids in the cell bodies of infected mouse dorsal root ganglia (DRG) [21,119]. This phenotype was also observed in vitro, in a biochemical system that reconstitutes kinesin and dynein-mediated trafficking of egressing HSV-1 virions along MTs; deletion of UL36 diminished the efficiency of HSV-1 binding to MTs, and reduced the frequency of viral trafficking along MTs by two thirds [22,120].

These data suggest that capsid-associated UL36p and UL37p are essential for the binding, or at least the activity, of MT-dependent molecular motors. These presumably include MT plus end-directed kinesins, which would be expected to support anterograde capsid transport to the cell periphery during egress (Section 1.2.1). The simplest model suggested by the above findings is that UL36p is required for capsid anterograde trafficking because it recruits UL37p [19,104,105,106,107,108]. UL37p then in turn directly, or indirectly, controls kinesin binding or activity. Alternatively, in addition to providing a UL37p-attachment function, UL36p might cooperate with UL37p to recruit or regulate kinesins. Mutational analysis of UL36p (Section 3.4) lends some support to this latter model, as do studies in PRV [121]. Fluorescently tagged PRV capsids egress through the Vero cell cytoplasm in a processive way, over distances of ~4–10 µm, at velocities of ~1–5 µm s^−1^ [121]. The MT-depolymerizing drug nocodazole abolished this processive curvilinear capsid transport, whereas cytochalasin D had no effect, suggesting motion was dependent upon MTs rather than F-actin [121]. As seen for HSV-1, a PRV strain deleted for UL36 exhibited only random, non-processive movement which, as would be expected, was unaffected by addition of nocodazole or cytochalasin D. Interestingly, unlike HSV-1, a PRV strain lacking UL37 exhibited some residual directional motion in the cytoplasm, though with reduced kinetics and processivity (typically less than ~5 µm). This suggests that PRV UL36p is able to recruit or activate kinesin on its own, and that UL37p supplies additional motor-recruitment roles or potentiates the function of UL36p [121]. This is discussed further in Section 3.4.

### 3.3. Kinesin Binding to Capsids In Vitro Requires Inner Tegument-Proteins 

Roles for UL36p and UL37p in kinesin attachment in vivo (Section 3.2), are consistent with biochemical studies in which purified HSV-1 capsids with varying tegument compositions were challenged to recruit molecular motors from purified motor preparations or crude brain cytosol [18]. Through a combination of immunoblots, quantitative mass spectrometry and quantitative immunoelectron microscopy it was found that capsids exposing the inner tegument proteins UL36p, UL37p and the US3p serine/threonine kinase (Table 1, [36,122]) were able to bind dynein, dynactin, and kinesin-1 (Figure 2). The ability to bind motors was lost if the inner tegument was absent, or occluded by the presence of the outer tegument [18]. HSV-1 capsids with exposed inner tegument were able to bind multiple copies of each motor, and bound plus and minus end-directed motors simultaneously, with a stoichiometry of approximately 0.3 to 60 copies of dynein, 0.5 to 80 copies of dynactin, and 2.5 to 380 copies of kinesin-1 per capsid. Purified kinesin-1, dynein and dynactin could also bind to purified capsids in the absence of other cellular proteins, suggesting they each interact directly with the inner tegument [18]. Interestingly, dynein and dynactin could bind independently of one another [18]. Thus, the HSV-1 capsid is an atypical dynein cargo in that it does not necessarily bind dynein in complex with its partner dynactin [123] (Figure 2).

The binding of dynein and dynactin to purified capsids in vitro [18] might be reconstituting the normal process of retrograde motor-recruitment to capsids that occurs during infection [76,77,78,79]. It has been known for some time that UL36p is the key effector recruiting dynein and dynactin to capsids during entry for both HSV-1 and PRV (Figure 2, reviewed in [6]). Dynein attaches to HSV-1 capsid vertices in the vicinity of UL36p [76,124], PRV UL36p coimmunoprecipitates with dynein and dynactin [80], and PRV UL36p can mediate retrograde transport of a surrogate cargo (mitochondria) to the cell periphery in the absence of other viral proteins [80]. However, even HSV-1 capsids undergoing anterograde transport during egress in the cytoplasm exhibit dynein-directed motion [21,119]. The mechanism of dynein recruitment during egress is less well understood and is considered in Section 3.5 and Section 4.5. 

### 3.4. Mechanisms of Recruitment of Kinesin Motors to Capsids via UL36p and UL37p 

Are there any clues in the sequence or structure of UL36p or UL37p that might point to the mechanism of kinesin motor recruitment? The Sodeik laboratory identified two “tryptophan-acidic” WD and WE motifs in UL36p that are conserved among all Alphaherpesvirinae. Alanine ablation of the motifs gave rise to HSV-1 strains with an approximately 3-log decrease in infectious titer and greatly impaired envelopment in the cytoplasm [19]. Capsids were also found to accumulate in a juxtanuclear region, lying over the microtubule organizing center (MTOC), with fewer capsids reaching the peripheral regions of the cell [19]. Importantly, the mutations did not result in gross structural changes to the surface of the egressing capsid; the mutant UL36p bound to capsids normally and supported recruitment of its binding partner UL37p, as well as the outer tegument polypeptides VP16 and VP22 [19] (Table 1, Figure 1, for a review of polypeptides in the outer tegument see [17]). The study concluded that the tryptophan-acidic motifs could play roles in UL36p conformational changes that support cytoplasmic capsid envelopment [19,20], an event for which UL36p is certainly critical [14,17,20,24]. Nevertheless, as the authors pointed out, WD/WE tryptophan-acidic motifs resemble the bipartite signals that mediate cargo protein-association with the TPR sequences of kinesin-1 KLCs (Section 1.2.1, Figure 2) [64,65,66]. Capsids bearing UL36p proteins with ablated WD/WE-motifs might therefore be unable to traffic from the MTOC to the site of cytoplasmic envelopment, and onward to the cell periphery, because they are unable to bind kinesin-1 and undergo MT-mediated anterograde traffic [19]. One prediction of this model is that these UL36p-mutant capsids should fail to bind kinesin-1 in vitro (Section 3.3), but this remains to be demonstrated.

If UL36p directly recruits kinesin-1, why does deletion of UL37p cause such a dramatic reduction in capsid anterograde transport for HSV-1 and (to a lesser extent) PRV? (Section 3.2, [21,119,121]). One model that would fit all of the available data is that UL36p WD/WE motifs directly recruit kinesin-1 motors by binding the TPR repeats of their KLCs, and UL37p stimulates the ability of UL36p to do so, perhaps driving UL36p conformational changes that make the WD/WE residues more accessible. This concept of modulation of UL36p-motor interaction by UL37p finds a striking parallel during viral entry. UL37-null PRV strains exhibit delayed trafficking of PRV capsids from the cell surface to the nucleus in rabbit kidney epithelial cells [125]. Furthermore, mutation of a conserved region termed R2, on the surface of UL37p, abolishes the ability of PRV and HSV-1 to invade the peripheral nervous system [28,126]. This defect arises from a striking “ping-pong” phenotype, wherein UL37p R2-mutant capsids exhibit back and forth anterograde/retrograde movement along MTs during entry, instead of normal, sustained, long-distance retrograde transport along axonal MTs to the cell body [126]. One explanation for this ping-pong motion is that the R2 region of UL37p normally suppresses a kinesin motor present on the retrograde capsid, allowing dynein-directed transport to dominate [126]. Taking all of these egress and entry observations together, UL37p may act as a “rheostat” for UL36p, turning its binding or activation of kinesin “down” during entry, to favor retrograde traffic, but “up” during egress in support of anterograde motion.

### 3.5. Dynein Recruitment by Alphaherpesvirus Capsids during Egress

It is increasingly clear that MT-directed transport of cellular cargo commonly involves bidirectional motion, and that the presence of multiple motors greatly enhances the processivity of transport, even when those motors act in opposition [127]. A complex process of balance and cross-talk between opposed motors determines the overall net direction of trafficking [128,129]. Similarly, alphaherpesviruses have long been known to exhibit bidirectional transport during overall anterograde traffic along the axon [6,130]. Furthermore, during HSV-1 egress in Vero cells and mouse DRGs, capsids move out to the cell periphery (Section 3.2), but also back from the periphery to the cell nucleus [21,119]. Both types of motion were abolished by deletion of UL36 and UL37, suggesting that UL36p/UL37p are essential for recruitment of not only kinesins, but also dynein, during egress [21,119] (Figure 1 and Figure 2).

Do capsids use UL36p to recruit dynein during egress, as they do during entry [76,77,78,79,80]? Yeast two-hybrid and pull-down studies suggest that, during egress, UL37p interacts with dystonin/bullous pemphigoid antigen 1 (BPAG1) [131,132] (Figure 1). Dystonin is thought to connect membrane-bound cargo to dynein/dynactin (Figure 1), and in its absence axonal transport of vesicles, multivesicular bodies (MVBs), mitochondria, and other organelles is impaired [133,134,135]. Dystonin also interacts with clathrin, the actin cytoskeleton, and influences MT stability and Golgi organization [131,136]. It is therefore possible that UL37p uses dystonin as a linker to recruit dynein to capsids during egress. The importance of dystonin for capsid transport was demonstrated by shRNA-knockdown in human fetal foreskin fibroblast 2 (HFFF2) cells [132]. In control HFFF2 cells, HSV-1 capsid trafficking was similar to that reported for HSV-1 and PRV in Vero cells [21,121], although for technical reasons HFFF2 studies were conducted at 25 °C rather than 37 °C; capsids exhibited both anterograde and retrograde transport, with an average travel distance of 5.6 µm and average velocity of ~0.95 µm s^−1^ [132]. However, following dystonin knock-down capsids showed essentially no motion (average travel distance of 0.54 µm and average velocity of ~0.19 µm s^−1^), resembling their behavior in the presence of nocodazole [132] or in the absence of UL36p or UL37p [21,121]. Why might dynein/dynactin be recruited by a UL37p-dystonin pathway, rather than UL36p, as during entry [76,77,78,79,80]? Perhaps evolution has selected UL36p to be as efficient a mediator of dynein-driven retrograde transport as possible, in order to maximize the efficiency of entry and the likelihood of infection. UL36p might therefore be less suitable than UL37p-dystonin for the more nuanced task of balancing kinesin and dynein motors during capsid egress.

Finally, although dynein recruitment during alphaherpesvirus anterograde transport is best understood in the context of the capsid, there is also evidence that dynein is present on PRV particles at later stages of export, following capsid envelopment in the cytoplasm [61] (Section 4.5). The mechanism by which dynein is recruited to these egressing virions is unknown.

## 4. Recruitment of Kinesin Motors to Organelles Transporting Enveloped Virions

### 4.1. Capsid Envelopment Transforms the Cargo That Must Be Transported during Alphaherpesvirus Egress

After MT-directed trafficking through the cytoplasm, the egressing alphaherpesvirus capsid associates with organelles derived from the *trans* Golgi network (TGN) or endosomes [12,15,17]. At this point, any remaining outer tegument components are recruited and the capsids bud, and pinch-off, to generate fully mature, enveloped particles within the organellar lumen [12,14,15,17]. These organelle-associated enveloped virions (OEVs) are larger and more massive than non-enveloped capsids, and unlike the rigid proteinaceous capsid-shell, the bounding lipid bilayer presents a flexible surface for motor attachment [6,17]. These substantial differences may well result in alphaherpesvirus capsids and OEVs selecting different sets of kinesin motors to accomplish their anterograde traffic. However, our understanding of why particular kinesins are selected for transport of a given cellular or viral cargo is extremely limited (Section 1.2.1). 

### 4.2. The gE/gI-US9p Complex Is Required for MT-Directed Transport into, or along, the Axon 

The mechanism of motor recruitment to the alphaherpesvirus OEV is best understood in infected neurons, where the virally encoded membrane glycoproteins E and I (gE and gI), and a non-glycosylated membrane protein encoded by the US9 gene (US9p) play critical roles in MT-directed anterograde traffic into, or along, the axon [101,137,138,139,140,141,142,143] (Table 1, Figure 1 and Figure 2). gE and gI are type I membrane proteins with 300- to 400-amino-acid long extracellular domains and 90- to 110-amino-acid long C-terminal cytoplasmic tails, and are thought to always function in the context of a gE/gI heterodimer [6,41,50,101,144,145] (Figure 2). US9p is a small (90 amino acid long in HSV-1 strain 17) lipid raft-associated type II membrane protein, with its C-terminal ~20 amino acids embedded in the lipid bilayer and the amino terminus of the protein directed towards the cytoplasm [49,50,146] (Figure 2). Interestingly, the genes encoding gI, gE and US9p (US7, US8 and US9 respectively) lie adjacent to one another in the alphaherpesvirus genome, with US7 and US8 exhibiting linkage disequilibrium, consistent with direct protein-protein contact between their gI and gE gene products [147] 

Although gE/gI and US9p are important for anterograde spread of both HSV-1 and PRV, there are differences in the phenotypes of PRV and HSV-1 strains lacking these membrane proteins. For HSV-1, the gE/gI and US9p proteins appear to function redundantly in support of anterograde transport [148,149,150,151]. Loss of gE/gI, or US9p, each reduces the numbers of HSV-1 particles reaching distal axons by 60–75%, but their simultaneous deletion abolishes virion delivery to axons almost completely [41,42,43]. For PRV, loss of gE/gI reduces particle delivery to the axon by ~75% [152], but deletion of US9p alone blocks essentially all virions from appearing in the axon, and abolishes the anterograde spread of infection [50,144,145,153]. Interestingly, the gE/gI heterodimer (though not US9p) is also required for efficient delivery of enveloped HSV-1 viruses to cell–cell junctions at the lateral surfaces of polarized epithelial cells, an important mechanism of cell–cell spread [101,154,155]. Whether gE/gI might direct HSV-1 particles to epithelial cell–cell junctions via MT-directed transport [17,101] is considered further in Section 4.5. 

### 4.3. Biochemical and Genetic Evidence Suggest That a gE/gI-US9p Complex Recruits the Kinesin-3 Motor KIF1A to the PRV OEV

To investigate the mechanism responsible for the striking axon-entry/transport deficit of PRV US9-null mutants, Kramer and colleagues used mass spectrometry to identify proteins that copurified with a GFP-US9p fusion in extracts of PRV infected PC12 cells [62]. Among the cellular proteins pulled-down by GFP-US9p was the kinesin-3 motor KIF1A [62] (Section 1.2.1). A mutant form of PRV US9p, in which the trans-membrane domain had been replaced by that of the transferrin receptor (TfR TMD-US9p), was unable to support PRV anterograde spread, and did not associate with KIF1A [62]. Similarly, tyrosine to alanine mutations in the cytoplasmically disposed amino-terminus of US9p gave rise to a mutant protein unable to pull down KIF1A [62], and that was severely limited in its ability to traffic PRV particles into the axons of cultured SCG’s [144] or to support PRV anterograde spread in a rat eye infection model [146]. Together these data suggest that US9p is a component of a KIF1A-recruitment mechanism for PRV. Consistent with this model, anterograde-, but not retrograde-, trafficking PRV particles were found to colocalize with KIF1A in axons of infected rat SCG neurons [62]. Furthermore, US9p (as well as gE/gI) were detectable in anterograde trafficking PRV OEVs in the axon, as expected if US9p recruits and/or regulates KIF1A for transport [156]. Furthermore, expression of a dominant negative KIF1A, in which the KIF1A N-terminal motor domain was substituted with GFP, resulted in a significant reduction in the numbers of anterograde-directed capsids in axons [62]. Finally, the axonal sorting, transport, and anterograde spread defects of a PRV strain lacking gE/gI and US9p could be corrected by artificially directing the recruitment of KIF1A to PRV OEVs using an inducible protein heterodimerization system [156]. Together, these data suggest that the critical requirement for gE/gI-US9p in axonal transport can be satisfied, or at least suppressed, by providing the KIF1A motor to the PRV OEV. 

If US9p is an essential component of a KIF1A recruitment apparatus on the PRV OEV, what is the role of gE/gI? The efficiency of pull-down of KIF1A by US9p was stimulated by the presence of gE/gI [152], suggesting gE/gI helps to stabilize a putative US9p-KIF1A complex, or affects the biological properties or localization of US9p [62,152]. One intriguing observation is that in the absence of gI (and thus presumably the gE/gI complex) PRV OEVs have reduced maximum velocities in the axon, and more frequent retrograde motility. This was interpreted to suggest that gE/gI might accelerate KIF1A movement or inhibit opposed dynein-mediated retrograde transport [156]. However, since gE/gI enhances the efficiency of US9p association with KIF1A [152] a simpler explanation is that, in the presence of gE/gI, each US9p molecule is more likely to recruit a KIF1A motor to the PRV OEV; larger numbers of attached kinesin motors can result in increased cargo velocities, depending upon the distribution of motors on the cargo surface and the load they encounter [157,158]. Despite the above findings however, evidence for assembly of a gE/gI-US9p-KIF1A complex has been elusive [62,156]. gE/gI, US9p and KIF1A do appear to coexist in detergent resistant membrane (DRM) “lipid rafts” isolated from US9p-null PRV-infected or uninfected SCG neurons [156]. In contrast, KIF1A was largely absent from DRMs when they were prepared from uninfected SCGs, SCGs infected by a US9p-null PRV [156], or SCGs infected by PRV expressing the TfR TMD-US9p mutant (see above) which fails to pull-down KIF1A [62]. These data are consistent with a model in which lipid rafts concentrate gE/gI and US9p, perhaps stabilizing formation of a tripartite gE/gI-US9p complex that then recruits KIF1A to raft-enriched regions of the bilayer surrounding the OEV [156] (Figure 2). Multi-channel super-resolution and live TIRF microscopy of PRV infected rat SCG neurons reveal that gE/gI, US9p and KIF1A become associated together in lipid rafts in the TGN [156], presumably reflecting capsid cytoplasmic envelopment and assembly of motile OEVs at that location (Section 4.1, reviewed in [17]). Interestingly, the lipid PI [4,5] P2, which helps recruit KIF1A to membranes by binding to its C-terminal PH domain (Section 1.2.1), is concentrated in cholesterol and sphingomyelin-rich lipid rafts, at least at the plasma membrane [159]. However, it is less clear whether rafts in the bilayer of the TGN, or other intracellular organelles, are enriched in this lipid. 

### 4.4. Evidence That KIF1A May Transport Alphaherpesvirus OEVs and Envelope Proteins from the Cell Body to the Axon

Data described in Section 4.2 and Section 4.3 are consistent with a model in which gE/gI-US9p serves to recruit KIF1A to the surface of OEVs for anterograde traffic along the axon [62,152]. However, other findings suggest this explanation is incomplete. Studies in infected chick DRGs confirmed that US9p-null PRV OEVS are rarely found in axons, but demonstrated that those few that are present exhibit transport kinetics indistinguishable from wild type OEVs [160]. Similar results were obtained for HSV-1 [41,42]. These data suggest that gE/gI- or US9p-null alphaherpesviruses are largely absent from the axons of infected neurons because they fail to enter them, not because of subsequent axonal trafficking defects [6,160]. If so, the primary function of gE/gI-US9p is to recruit KIF1A for transport of alphaherpesvirus OEVs from the cell body into the axon [160]. For HSV-1 it has also been proposed that gE/gI-US9p are required for efficient envelopment of capsids in the cell body [41]. Thus, in the absence of gE/gI and US9p fewer HSV-1 OEVs would be assembled and available for transport, explaining why diminished numbers are present in the axon [41,161,162].

Membrane-imbedded alphaherpesvirus envelope proteins traffic within neurons as constituents of OEVs, but are also carried separately from virions, within transport vesicles [6,50,144]. US9p and KIF1A appear to be important for export of these vesicles from the cell body, since deletion of US9 results in failure of most PRV envelope proteins to enter the axon [50,144,145,163]. Nevertheless, anterograde MT-directed transport of at least one PRV encoded glycoprotein, gM, is not US9p-dependent [163]. HSV-1 glycoproteins show variable dependence upon gE/gI and US9p for delivery to the axon [6,43,164].

### 4.5. Which Kinesins Support Alphaherpesvirus Transport along the Axon, and How Are They Recruited? 

**Figure 3:**
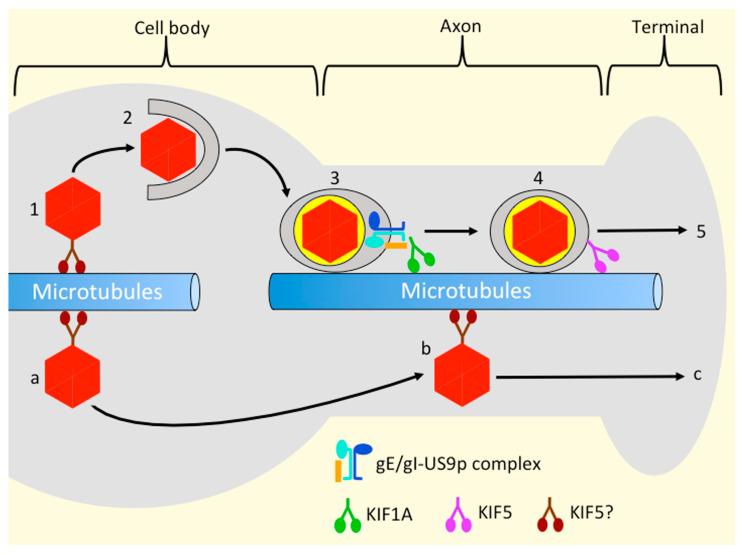
The Married and Separate models for alphaherpesvirus egress in neurons, and possible roles for kinesins KIF1A and KIF5 during transport. Steps 1–5: Married model. (**1**) After emerging from the nucleus, packaged capsids (red hexagons) recruit kinesin motors (shown in brown, possibly isoforms of KIF5 as detailed in Figure 2a to traffic through the cytoplasm of the cell body). (**2**) Upon reaching their site of envelopment, capsids bud into the organellar lumen to generate a mature OEV. (**3**) The OEV (depicted as in Figure 2b utilizes the gE/gI-US9p apparatus (blue lollipops and orange bar) to recruit KIF1A (shown in green), and ensure its delivery from the cell body into the axon. (**4**) Upon reaching the axon, the OEV may recruit isoforms of KIF5 (shown in purple) for subsequent transport, and either retains KIF1A or targets it for destruction (see text for details). (**5**) Kinesin-mediated transport eventually delivers the OEV to the nerve terminal for subsequent exocytosis and spread. Steps (**a**–**c**): Separate model. (**a**) Capsids utilize kinesin motors and MTs to travel through the cell body cytoplasm and into the axon. (**b**) Non-enveloped capsids travel along the axon, though the identity of the kinesin(s) used at this stage remains unknown. (**c**) After arriving at the nerve terminal, the capsid acquires its envelope by a process similar to that in step 2, and infectious virions are released by exocytosis.

If gE/gI-US9p-KIF1A ensures delivery of OEVs into the axon, which kinesin motors transport them along axonal MTs, and to the nerve terminal? In the case of PRV it is possible that KIF1A also mediates this step, since KIF1A is present on PRV OEVs during their axonal transport [62]. If this is the case then, for reasons discussed in Section 4.4, the KIF1A motors that drive axonal trafficking must be recruited to OEVs independently of gE/gI and US9p. Alternatively, we found that a subpopulation of egressing PRV OEVs were decorated with the neuronal-specific kinesin-1 isoform KIF5C (Section 1.2.1) in infected, differentiated, mouse neuronal CAD cells [61]. We also observed KIF1A-associated PRV OEVs, but KIF5C and KIF1A were never present simultaneously on the same viral particle, suggesting these two motors bind egressing virions at distinct stages of export [61]. Interestingly, KIF5C association with egressing PRV only occurred in differentiated CAD cells, whereas KIF1A binding occurred in both differentiated and undifferentiated CAD cells [61]. We interpreted our data to suggest that the function of KIF1A is to sort PRV OEVs from the cell body to a differentiated-neuron specific location, such as the axon or axon initial segment [165], where KIF5C or other kinesin-1 motors can be loaded onto the particle for transport down the axon [6,61]. Deletion of the gE/gI-US9p complex prevented KIF1A binding to OEVs, as expected, but also eliminated recruitment of KIF5C [61]. This is consistent with a model in which gE/gI-US9p-KIF1A is essential for trafficking of virions to the KIF5C differentiation-dependent loading site, as summarized in Figure 3.

A recent study may shed additional light on how KIF1A and KIF5C motors might be exchanged during egress. Upon PRV infection of rat PC12 cells and SCG neurons, KIF1A becomes targeted for degradation by the proteasome, and gE/gI-US9p are sufficient to induce this accelerated turnover in the absence of other PRV proteins [166]. As pointed out by Huang and colleagues [166], ubiquitin-mediated degradation of KIF1A has been reported in *Caenorhabditis elegans* where it functions to remove KIF1A from its cargo after delivery of synaptic vesicles to the synapse [167]. Presumably the neuron finds it more efficient to destroy motors at the end of their long journey to the synapse, than to separate them from their cargo and return them to the cell body for additional rounds of transport. Degradation of kinesin-1 and the kinesin-2 KIF3A were not accelerated by PRV infection [166]. Huang and colleagues concluded that their data are consistent with KIF1A playing a predominant role in PRV transport along the axon [62]. However, it may be that this is the mechanism by which KIF1A is efficiently removed prior to KIF5C loading, and would explain why KIF1A and KIF5C are never seen on the same PRV OEV [61] (Figure 3).

Deletion of gE/gI-US9p, with concomitant loss of KIF1A and KIF5C, dramatically altered the trafficking properties of PRV OEVs in an in vitro MT-dependent transport assay. gE/gI-US9p-null OEVs were diminished in their ability to traffic to the plus ends of MTs, and exhibited a greater frequency of minus end-directed and bidirectional movement [61]. This indicates that OEV-bound KIF1A and/or KIF5C are engaged in, and normally win, a “tug of war” against opposed dynein motors, and immunocytochemistry confirmed the presence of dynein on the egressing OEVs [61]. gE/gI-US9p-null PRV OEVs isolated from porcine kidney epithelial PK15 cells showed a similar in vitro trafficking defect [61]. It is well established that gE/gI (though not US9p [141]) help ensure cell–cell spread of infection by delivering alphaherpesviruses, including HSV-1 and PRV [17,101,155,168], to the lateral surfaces and cell–cell junctions of polarized epithelial cells [101,154]. Our in vitro data suggest that one mechanism by which this may be achieved in epithelia is to recruit kinesins, ensuring processive MT plus-end directed traffic to the cell periphery [61].

Several other lines of evidence point to a role for kinesin-1 motors in the transport of HSV-1 OEVs. Following HSV-1 infection of CAD cells, egressing axonal HSV-1 particles colocalized with KIF5C, but not with KIF1A [63] (Figure 3). Moreover, RNAi-mediated silencing of each of the three kinesin-1 KHC isoforms KIF5A, -5B, and -5C [53,54,55] (Section 1.2.1), or of KLCs [54], inhibited most HSV-1 transport in axons, whereas silencing of KIF1A had little effect [63]. Furthermore, in HSV-1 infected human fetal DRGs, immunogold electron microscopy indicated the presence of kinesin-1 on OEVs in axons, axonal varicosities and growth cones [169], though the kinesin-1 isoform was not identified in that study.

How might kinesin-1 motors be recruited to egressing alphaherpesviruses? One possibility is the amyloid precursor protein (APP), a type-1 membrane protein found in axonal transport vesicles and implicated in the kinesin-1-dependent transport of cargo proteins including presenilin 1, Growth associated protein 43 (GAP-43), synapsin-I and Tropomyosin receptor kinase A (TrkA) [170,171]. Anterograde-trafficking HSV-1 OEVs have been found in association with APP in a squid axon model of transport [172] and in CAD neurons [63]. However, interpretation of this finding is complicated by the fact that the biology of APP is not well understood, and its role as a kinesin-1 receptor is far from resolved [56,173]. In other studies, the kinesin-1 KHC was used to pull-down putative kinesin-1 receptors from HSV-1 infected HEp-2 cells [174]; candidates included the major capsid protein VP5 and the tegument proteins US11, VP22 and VP16 [17,174] (Table 1). Similar approaches led to the suggestion that the C-terminal tail of KIF5B interacts with US9p [52]. However, the significance of these observations for kinesin-1 recruitment during HSV-1 egress in vivo remain to be determined. Furthermore, if US9p is essential for kinesin-1 recruitment during axonal transport then deletion of the US9 gene should affect the trafficking of HSV-1 particles that are able to reach the axon. This does not appear to be the case [41,42].

### 4.6. Kinesin Recruitment and the Married and Separate Mechanisms of HSV-1 Transport

Any understanding of kinesin recruitment during alphaherpesvirus egress must account for the fact that, in some types of neurons, it has been proposed that non-enveloped HSV-1 capsids, rather than OEVs, traffic out of the cell body and along axons. These capsids eventually reach, and acquire their envelopes, at the nerve terminal. This has been termed the “Separate” model of transport, in contrast to the “Married” model where the capsid and envelope unite in the cell body then travel together in the context of the OEV (Figure 3). It is generally agreed that PRV exclusively utilizes the Married mechanism for axonal transport, but for HSV-1 the question of Married or Separate is a matter of debate [41,142,161,175,176,177,178,179]. This issue has recently been summarized [6], and lies beyond the scope of the current review. However, it is worthwhile to consider the implications of the Married and Separate mechanisms for kinesin recruitment during egress. We speculate that, in the Separate mechanism, HSV-1 capsid transport along the axon utilizes kinesin-1 motors, recruited to the capsid surface by the same inner tegument UL36p/UL37p-mechanism used in the cell bodies of Vero cells and mouse DRGs [21,119] (Section 3.2, Section 3.3 and Section 3.4), as shown in Figure 3. A more challenging problem is how the integral membrane proteins gE/gI and US9p might influence kinesin recruitment to non-enveloped capsids. One hypothesis is that gE/gI-US9p, and associated tegument proteins on the cytoplasmic surface of cell-body organelles, provide a platform that “loads” kinesins onto capsids for delivery to the axon [42,43,161,162].

### 4.7. UL56p May Play Roles in Kinesin Binding and Virus Envelopment

The alphaherpesvirus UL56p gene product [180] (Table 1, Figure 1) bears some similarity to US9p in that it is a type II, membrane-anchored phosphoprotein that partitions into lipid rafts and may associate with kinesin KIF1A [62,181]. UL56p is largely dispensable for viral replication in tissue culture [182], but facilitates transport of HSV-2 out of cultured epithelial cells [34] and is important for HSV-1 pathogenesis [183]. Similarly, although deletion of UL56 from PRV has no impact on anterograde transport of virus in vivo, it does result in attenuated virulence in a mouse intranasal infection model [182].

In Vero cells, the UL56p of HSV-2 localizes to juxtanuclear vesicular structures resembling MVBs, late endosomes or lysosomes [35,180], and in this location binds and targets for destruction the E3 ubiquitin ligases Nedd4 and Itch [33,34,35]. Intriguingly, Nedd4 and Itch are Homologous to E6AP C-terminus (HECT) ubiquitin ligases that play critical roles in the function of the cellular Endosomal Sorting Complex Required for Transport (ESCRT) machinery [14,184,185,186]. Proteins ubiquitinated by HECT ligases subsequently interact with components of the ESCRT apparatus, which direct them into assembling MVBs for proteolytic degradation [187]. It is unclear why HSV-2 utilizes UL56p to destroy MVB-associated Nedd4 and Itch, but one possibility is that removal of these ubiquitin ligases would deplete the MVB of ubiquitylated proteins, molecules that might otherwise compete with viral capsids for access to the ESCRT apparatus during envelopment [14,188]. Localization of UL56p to sites of envelopment could also be used to recruit KIF1A to those organelles, either for transport of the resulting OEV to the periphery, or to oppose the normal process of dynein-mediated retrograde traffic of MVBs, that would otherwise deliver OEVs to the lysosome [189,190].

## 5. Myosin-Actin Transport of Alphaherpesviruses during Late Stages of Egress

Myosin motors have been implicated in the release of alphaherpesviruses from infected cells, and their spread to uninfected cells, though few molecular details are known [191]. One mechanism of myosin-mediated spread may be via TNTs (Section 1.2.3), 10–100 µm long, thin membranous processes that normally transport ions, proteins and organelles between cells [89,192,193] and which have been coopted as a means of spread by many virus families [194]. Infection by HSV-1 [17,31,32,195], and several other alphaherpesviruses [17,39,194,196], induce TNT formation in a process dependent upon the virally expressed US3p serine/threonine kinase [38,194,197,198] (Table 1). TNTs may provide channels for direct transport of virus from infected cells to the cytoplasm of neighboring target cells [199], however it has also been observed that viral particles can be released from the entire length of the TNT, not just at the TNT/target-cell junction [199]. This suggests that one function of TNTs is to increase the number and proximity of contact points between infected and uninfected cells, ensuring efficient transmission of extracellular enveloped virions [199].

If TNTs facilitate alphaherpesvirus spread from infected to uninfected cells, how might viral particles move along them? TNTs extending from both uninfected and alphaherpesvirus-infected cells commonly contain F-actin [89,192,193]. Furthermore, at least in uninfected cells, TNTs contain the unconventional myosins (Section 1.2.3) myosin-X, involved in TNT assembly [200], and myosin-Va, which may drive intra-TNT trafficking of endocytic organelles [88,89]. TNTs of diameter greater than ~0.7 µm can also contain MTs [38,194,196], that may be stabilized by acetylation and detyrosination [199]. It is therefore possible that alphaherpesvirus particles recruit myosins or kinesins for transport along TNTs.

Consistent with a role for myosins in TNT-mediated alphaherpesvirus egress, HSV-1 infection of Vero cells induces the formation of TNT-like structures that contain myosin NM-IIA [32] (Section 1.2.3). This TNT NM-IIA colocalized with puncta containing a GFP fusion to the outer tegument protein VP22 [17,32] (Table 1, Figure 1), and an anti-VP22 affinity column pulled down both VP22 and NM-IIA from extracts of HSV-1 infected HeLa cells. GFP-VP22 was also observed to colocalize with NM-IIA in juxtanuclear structures presumed to correspond to the Golgi apparatus, and also at cell–cell junctions [32]. However, the relationship between these structures, TNTs and HSV-1 egress is unclear. The general myosin inhibitor BDM (Section 2) inhibited release of HSV-1 to the media 20–50 fold, with little effect upon yields of cell-associated virus [32], but whether BDM affected HSV-1 entry into, or along, the TNT-like processes was not determined. Similar TNT-like projections, reaching from HSV-1 infected Vero cells to adjacent uninfected cells, contained puncta exhibiting GFP-VP16 fluorescence [31] (Table 1, Figure 1). These VP22 and VP16 outer tegument protein-containing foci could correspond to NM-II-bound egressing HSV-1 particles, either tegument-associated capsids (Section 3) or OEVs, though PRV-induced TNTs contained only OEVs [39,194,199]. Whatever the nature of the trafficking virion, nothing is known of how egressing alphaherpesvirus particles might recruit NM-IIA for transport within a TNT. Moreover, most studies of TNT assembly and function during alphaherpesvirus infection have made use of immortalized cell lines. TNT-mediated transmission of bovine herpesvirus 1 (BoHV-1) has been observed in primary calf skin fibroblasts [196], and the US3 gene of PRV is sufficient to induce formation of TNT-like structures in mouse embryonic fibroblasts [201], however the significance of TNTs for alphaherpesvirus spread between primary epithelial cells and neurons is unclear.

Myosin-Va has also been implicated in HSV-1 egress from infected cells, though whether this is mediated via TNTs remains to be determined. HSV-1 infection of HEp-2 and HeLa cells enhanced the immunoreactivity of a particular myosin-Va epitope without changing overall levels of the myosin-Va protein [202], suggesting HSV-1 infection was driving myosin-Va conformational changes that might accompany motor activation [86,202]. Similarly, the secretion of HSV-1 from infected HeLa cells was reduced approximately four-fold, and cell-associated infectivity slightly increased, by a dominant negative allele of myosin-Va [202].

## 6. Conclusions

Alphaherpesvirus trafficking within and between cells is critical for viral replication and the spread of disease, and is accomplished by the conscription and exploitation of a variety of cellular motors. The best understood example of this, by far, is kinesin KIF1A recruitment by the gE/gI-US9p complex of PRV. However, even here we lack fundamental molecular details. Is the short cytoplasmic tail of US9p a stoichiometric receptor that directly contacts KIF1A, or does it act catalytically to stimulate KIF1A binding to, and dimerization on, the surface of the OEV bilayer? What is the role of gE/gI, and how is KIF1A recruitment coordinated with that of other kinesins, such as kinesin-1? As discussed above, the presence of dynein on egressing capsids and OEVs may enhance the processivity of transport during egress, but direct evidence for this is lacking. Furthermore, although there are some clues as to how dynein may bind to capsids during egress, the mechanism of dynein association with OEVs, and how egressing virions might coordinate dynein and kinesin activity to ensure processive anterograde transport, remain completely unknown. A fascinating area of study, still in its infancy, is the mechanism by which myosin is recruited by these viruses. It appears clear that alphaherpesvirus transport along TNTs is a significant route for alphaherpesvirus cell–cell spread, which may involve TNT actin filaments and virally associated myosin NM-IIA or Va. However, we know very little of how egressing alphaherpesviruses enter TNTs, and how they might select and bind myosins for transport. Other outstanding key questions are as follows.

- What is the advantage of selecting kinesin-1 and kinesin-3 for transport, and do these motors indeed traffic alphaherpesviruses at distinct stages of egress, as discussed above? How does the putative gE/gI-US9p-KIF1A complex deliver viral particles from the neuronal cell body to the privileged environment of the axon?
- Are the tryptophan acidic WD/WE motifs in UL36p required for kinesin-1 binding to capsids in vitro, and are they needed for processive, anterograde traffic of capsids in the cytoplasm prior to capsid envelopment?
- Does kinesin recruitment by gE/gI play a role in delivery of alphaherpesviruses to the lateral surfaces of polarized epithelial cells during cell–cell spread?
- Do gE/gI and US9p mediate traffic along TNTs that contain MTs?

## Figures and Tables

**Table 1:** Alphaherpesvirus-encoded proteins discussed in this review.

Protein Name (Alternate Name)	Location	Functions ^1^	Section ^2^ [Key References]
UL36p (VP1/2)	Inner tegument.	Foundation for recruitment of outer tegument via VP16. Binds UL37p. Cooperates with UL37p to recruit kinesin-1 to the capsid. Envelopment.	3 [17,18,19,20,21,22,23,24]
UL37p	Inner tegument.	Binds UL36p, dystonin. Cooperates with UL36p to recruit kinesin-1 to the capsid. Envelopment.	3 [17,21,25,26,27,28,29,30]
UL48p (VP16)	Outer tegument.	Connects UL36p to outer tegument. Found in foci located in TNTs.	5 [17,31]
UL49p (VP22)	Outer tegument.	Found in foci located in TNTs. Binds to NM-II.	5 [17,32]
UL56p	Envelope/membrane of cytoplasmic organelles.	Virulence. May bind KIF1A. Targets E3 ubiquitin ligases Nedd4 and Itch for destruction.	4.7 [33,34,35]
US3p	Inner tegument.	Serine/threonine kinase. Assembly of TNTs.	5 [17,36,37,38,39,40]
gE/gI heterodimer	Envelope/membrane of cytoplasmic organelles.	Trafficking of virions into or along axons. Sorting to epithelial cell–cell junctions. Facilitate HSV-1 envelopment in neurons.	4.2–4.6 [17,41,42,43,44,45,46,47,48]
US9p	Envelope/membrane of cytoplasmic organelles.	Trafficking of virions into or along axons. Facilitates HSV-1 envelopment in neurons.	4.2–4.6 [17,49,50,51,52]

**^1^** Limited to functions relevant to this review. ^2^ Section(s) of this review where the protein is discussed.

## Data Availability

Not applicable.

## Abbreviations

ESCRT	Endosomal sorting complex required for transport
HSV-1	Herpes simplex virus type 1
KHC	Kinesin heavy chain
KLC	Kinesin light chain
MT	Microtubule
MVB	Multivesicular body
OEV	Organelle-associated enveloped virion
PH	Pleckstrin homology domain
PRV	Pseudorabies virus (suid alphaherpesvirus 1)
TGN	*trans* Golgi network
TNT	Tunneling nanotube
ULnumber	Unique long (position of gene in HSV-1 genome)
USnumber	Unique short (position of gene in HSV-1 genome)
VPnumber	Virus Protein
